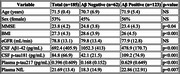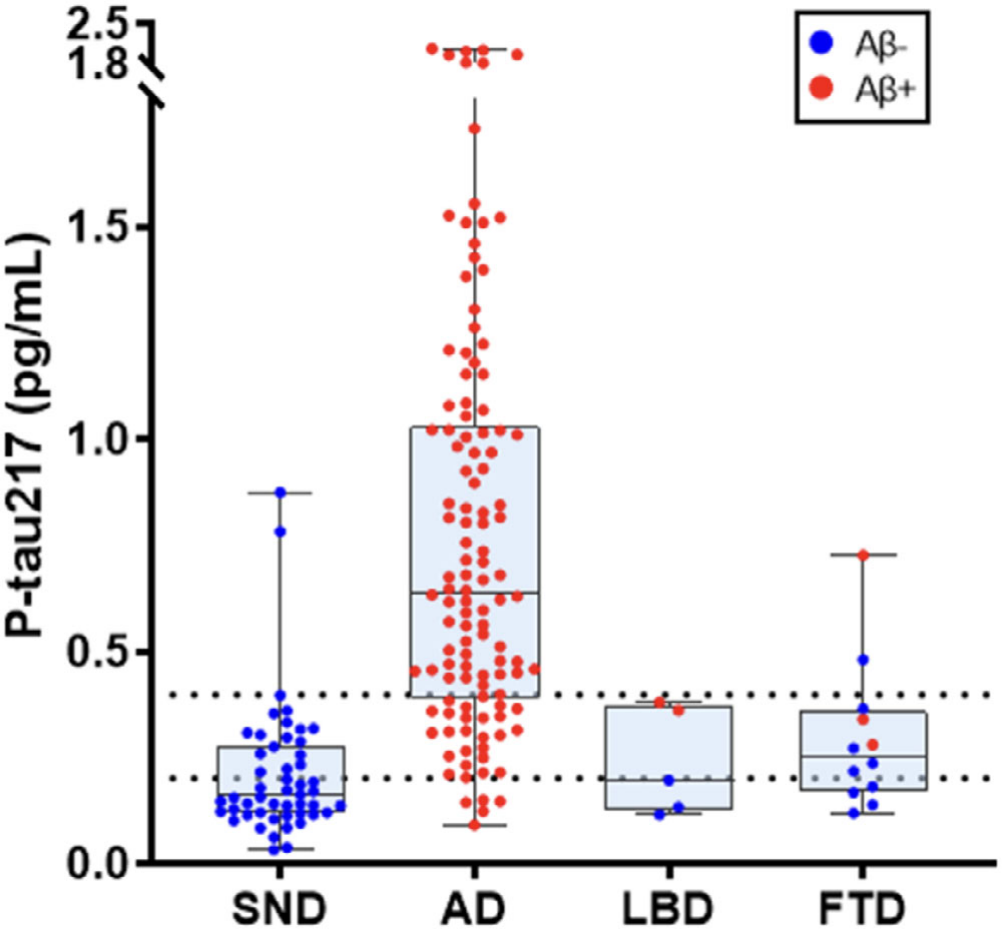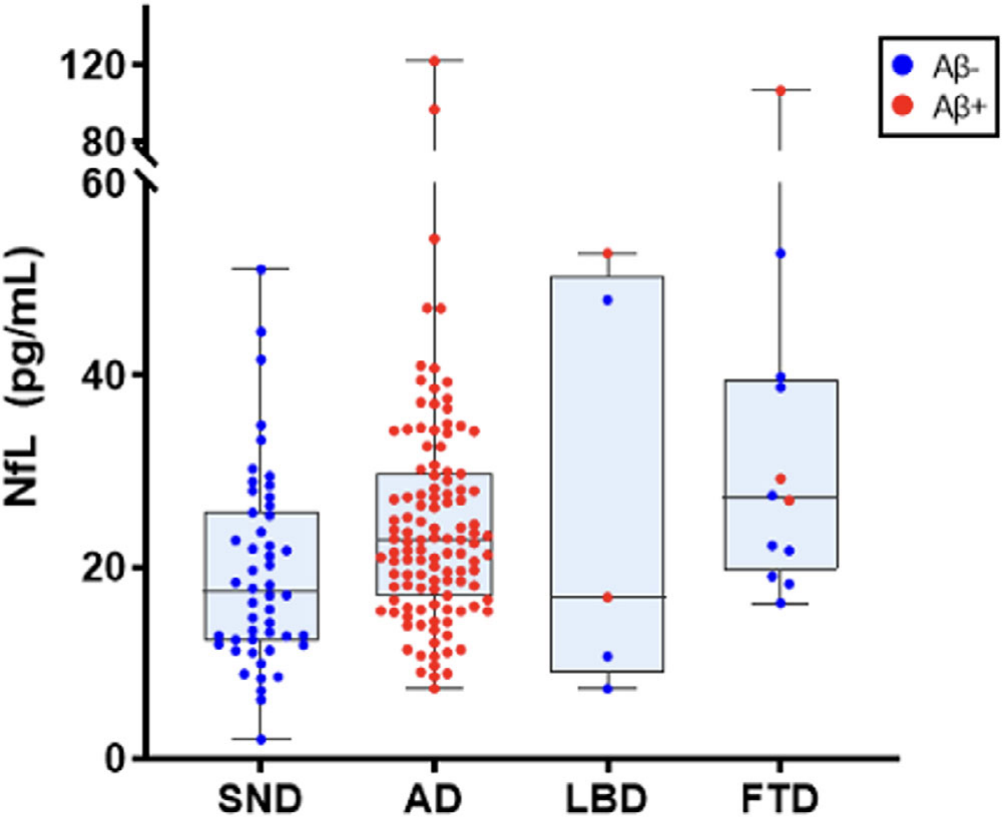# Utility of Plasma pTau217 and NF‐L in Predicting β‐Amyloid Status: Findings from a Multicenter Study in Catalonia

**DOI:** 10.1002/alz70856_101518

**Published:** 2025-12-25

**Authors:** Miquel Massons, Jordi Sarto, Núria Guillén, Neus Falgàs Martínez, Sergi Borrego‐Écija, Magdalena Castellví, Adrià Tort‐Merino, Anna Antonell, Roger Puey, Guadalupe Fernandez‐Villullas, Josep Maria Augé, Sara de la Fuente Olanda, Anna Colmenero, Gerard Piñol‐Ripoll, Iolanda Riba Llena, Anna Carnes, Marta Culell Juncà, Maira Teresa Osuna Pulido, Lorena Bajo Peñas, Teresa Romero, Eva Bonjoch Jaques, Joan Bello López, Susana Fernández González, Marta Balagué Marmaña, Isabel Gómez‐Ruiz, Anuncia Boltes Alandí, Claustre Pont Sunyer, Raquel Cuevas, Sara Carrillo, Laura Iglesias, Teresa Casadevall, Lorena Grau Guinea, Fernando Jose Espada Olivan, Raquel Sánchez‐Valle, Mircea Balasa, Albert Lladó, Alzheimer's disease and other cognitive disorders Unit. Hospital Clínic de Barcelona. Fundació de Recerca Clínic Barcelona – IDIBAPS. University of Barcelona, Barcelona, Spain

**Affiliations:** ^1^ Alzheimer's disease and other cognitive disorders Unit. Hospital Clínic de Barcelona. Fundació de Recerca Clínic Barcelona – IDIBAPS. University of Barcelona, Barcelona, Spain; ^2^ Alzheimer's disease and other cognitive disorders Unit. Hospital Clínic de Barcelona. Fundació de Recerca Clínic Barcelona – IDIBAPS. University of Barcelona, Barcelona, Barcelona, Spain; ^3^ Alzheimer's disease and other cognitive disorders Group. Service of Neurology, Hospital Clínic de Barcelona. Fundació Recerca Clínic Barcelona‐IDIBAPS, Barcelona, Spain; ^4^ Alzheimer's Disease and Other Cognitive Disorders Unit, Neurology Department, Hospital Clinic, Barcelona, Spain; ^5^ Unitat Trastorns Cognitius, Clinical Neuroscience Research, Santa Maria University Hospital, IRBLleida, Lleida, Spain; ^6^ Hospital Universitari Santa Maria de Lleida, IRBLleida, Lleida, Spain; ^7^ Hospital de Figueres. Fundació Salut Empordà, Figueres, Spain; ^8^ Fundació Hospital de la Santa Creu de Vic, Vic, Spain; ^9^ Hospital Moisés Broggi‐Consorci Sanitari Integral, Sant Joan Despí, Spain; ^10^ Fundació Privada Hospital Asil de Granollers, Granollers, Spain; ^11^ Hospital Sant Jaume de Calella. Corporació de Salut del Maresme i la Selva, Calella, Spain

## Abstract

**Background:**

Alzheimer's disease (AD) is the most common neurodegenerative dementia. Recent advancements in plasma biomarkers measuring phosphorylated tau (pTau), particularly pTau217, offer non‐invasive, cost‐effective alternatives to cerebrospinal fluid (CSF) and PET biomarkers for diagnosing AD. Plasma neurofilament light chain (NF‐L) has also emerged as a potential marker of neurodegeneration. This multicenter study evaluated the utility of these biomarkers as diagnostic tools and explored their potential to facilitate implementation across multiple centers, ensuring diagnostic uniformity.

**Method:**

Patients with mild cognitive impairment or mild dementia were recruited in a multicenter study (seven hospitals in Catalonia). Plasma levels of pTau217 and NF‐L were measured using Lumipulse G (Fujirebio). Their diagnostic accuracy was compared to established CSF biomarkers. Additionally, the influence of demographic factors such as age, sex, BMI, and glomerular filtration rate (GFR) on pTau217 levels was analyzed.

**Result:**

Of 212 enrolled patients, 27 were excluded due to pre‐analytical complications. Therefore, 185 patients were analyzed [Table 1], including 50 non‐neurodegenerative cases (SND), 119 AD cases, 5 Lewy body dementia cases (3 LBD Aβ‐, 2 LBD Aβ+), and 12 frontotemporal dementia cases (9 FTD Aβ‐, 3 FTD Aβ+). Plasma pTau217 detected amyloid pathology (Aβ+) with high accuracy using two cut‐offs: a cut‐off below 0.201 pg/mL yielded a sensitivity of 95%, with a negative predictive value of 87%. For values above 0.397 pg/mL, specificity reached 95%, with a positive predictive value of 97% [Figure 1]. These thresholds predicted β‐amyloid status in 74% of cases. NF‐L levels were elevated in neurodegenerative conditions with significant differences, but with low diagnostic precision (mean values: 26.434 vs 19.933 pg/mL, *p* = 0.01, AUC=0.634) [Figure 2]. Demographic factors, particularly BMI and GFR, had minimal effects on plasma biomarker levels, with limited impact on diagnostic performance.

**Conclusion:**

Plasma pTau217 showed high AD diagnostic accuracy, and may reduce the need for other procedures like CSF/PET. The use of two diagnostic thresholds appears optimal, with 26% of subjects in intermediate ranges, potentially indicating early AD changes. NF‐L were elevated in neurodegeneration but showed limited clinical utility in our study. This study supports the use of pTau217 in clinical practice, promoting diagnostic uniformity across Catalonia.